# Extensive morphological and immunohistochemical characterization in myotubular myopathy

**DOI:** 10.1002/brb3.147

**Published:** 2013-06-19

**Authors:** Minobu Shichiji, Valérie Biancalana, Michel Fardeau, Jean-Yves Hogrel, Makiko Osawa, Jocelyn Laporte, Norma Beatriz Romero

**Affiliations:** 1 Unité de Morphologie Neuromusculaire, Institut de MyologieGHU La Pitié-Salpêtrière, Paris, France; 2 Department of Pediatrics, Tokyo Woman's Medical UniversityTokyo, Japan; 3 Department of Translational Medecine, Institut de Génétique et de Biologie Moléculaire et Cellulaire (IGBMC), CNRS, UMR7104, INSERM, U964, Collège de France, Université de StrasbourgIllkirch, France; 4 Laboratoire Diagnostic Génétique, Faculté de Médecine – CHRUStrasbourg, France; 5 Groupe Hospitalier-Universitaire La Pitié-Salpêtrière, AP-HP, Centre de référence des maladies neuromusculairesParis-Est, Paris, France; 6 UPMC-Paris6 UR76, INSERM UMR974, CNRS UMR 7215, Institut de MyologieGHU La Pitié-Salpêtrière, Paris, France

**Keywords:** Centronuclear myopathies, *MTM1*, myotubular myopathy, myotubularin, satellite cells, T-tubules

## Abstract

The X-linked myotubular myopathy (XLMTM) also called X-linked centronuclear myopathy is a rare congenital myopathy due to mutations in the *MTM1* gene encoding myotubularin. The disease gives rise to a severe muscle weakness in males at birth. The main muscle morphological characteristics (significant number of small muscle fibers with centralized nuclei and type 1 fiber predominance) are usually documented, but the sequence of formation and maintenance of this particular morphological pattern has not been extensively characterized in humans. In this study, we perform a reevaluation of morphological changes in skeletal muscle biopsies in severe XLMTM. We correlate the pathologic features observed in the muscle biopsies of 15 newborns with *MTM1*-mutations according to the “adjusted-age” at the time of muscle biopsy, focusing on sequential analysis in the early period of the life (from 34 weeks of gestation to 3 months of age). We found a similar morphological pattern throughout the period analyzed; the proportion of myofibers with central nuclei was high in all muscle biopsies, independently of the muscle type, the age of the newborns at time of biopsy and the specific *MTM1* mutation. We did not observe a period free of morphological abnormalities in human skeletal muscle as observed in myotubularin-deficient mouse models. In addition, this study demonstrated some features of delayed maturation of the muscle fibers without any increase in the number of satellite cells, associated with a marked disorganization of the muscle T-tubules and cytoskeletal network in the skeletal muscle fibers.

## Introduction

The X-linked neonatal form of myotubular myopathy (XLMTM, OMIM 310400) is the most severe form of centronuclear myopathy. The disease is caused by mutations in the *MTM1* gene encoding myotubularin (MTM1) (Laporte et al. 1996). The severe neonatal form is characterized by hypotonia, muscle weakness, hypotrophy, and respiratory failure requiring assisted ventilation immediately after birth. The early survival can be compromised in male infants. Nonetheless some patients can, sometimes with severe disabilities, survive into childhood, adolescence, and even adulthood (Biancalana et al. 2003; Bertini et al. 2004; Hoffjan et al. 2006; Tosch et al. 2010). Survival rate may differ in various countries (McEntagart et al. 2002). The incidence of molecularly confirmed XLMTM is estimated at 1/100,000 male births per year (Biancalana et al. 2012).

Muscle biopsies are characterized by a large number of small muscle fibers with central nuclei resembling myotubes and a type 1 fiber predominance (reviews in Fardeau 1982; North 2004; Romero 2010; Romero and Bitoun 2011). Since these morphological findings resemble an early stage of fetal muscle development, myotubular myopathy has been proposed to result from an arrest in myogenesis (Sarnat 1990). In contrast to XLMTM newborn patients, the *Mtmt1*-null mice show no symptoms at birth but develop a progressive muscle disorder starting in the hind limbs at 3–4 weeks of age, leading to severe generalized amyotrophy and early death by about 7–12 weeks of age (Buj-Bello et al. 2002). Several weeks after birth the *Mtm1*-null mice begin to show the same histological features observed in XLMTM newborn patients, with muscle fiber atrophy, an increased proportion of type 1 fibers and centralization of nuclei. As muscle differentiation and maturation in *Mtm1*-null mice appears normal during the early stages of life, it has been proposed that defects in maintenance of muscle cell architecture might be responsible for the centralization of nuclei and mislocalization of organelles. Defects in triad structure and in calcium homeostasis may play an important physiopathological role in myotubular myopathy (Al-Qusairi et al. 2009; Dowling et al. 2009; Al-Qusairi and Laporte 2011; Toussaint et al. 2011).

The aim of our present study was to characterize the exact sequence of pathological events which occur in newborn infants with myotubular myopathy. Therefore, (a) We have reevaluated the morphological features of skeletal muscle biopsies according to the “adjusted-age” or age of XLMTM newborns at the time of the muscle biopsy; (b) We have analyzed the findings in muscle biopsies taken from two different territories (vastus lateralis and deltoid); (c) We have analyzed the progression of this myopathy using appropriate markers to assess the chronology of skeletal muscle development.

## Material and Methods

### Patients

Fifteen newborn infants with genetically characterized severe myotubular myopathy were enrolled. Patients 8 and 9 are brothers (Table 1). Eight of the 15 XLMTM patients are described for the first time and for seven of the 15 patients the molecular defects have been reported previously (Table 2) (Laporte et al. 1997; Buj-Bello et al. 1999; Biancalana et al. 2003). Prenatal diagnosis was not made for any patient. At birth, the gestational age of newborns ranged from 31 to 42 weeks of gestation. Six boys (Patients 1–6) were born prematurely, between 31 and 36 weeks of gestation, and 9 boys (Patients 7–15) were born at full term, from 38 to 42 weeks of gestation (Table 1). Muscle biopsies from vastus lateralis (*n* = 4) and deltoid (*n* = 3) muscles of seven individuals with no neuromuscular disorder were used as controls. The “adjusted-age” at muscle biopsy ranged from 37 weeks of gestation to 3 months of age.

**Table 1:** Summary of clinical features and muscle biopsy findings

	Preterm newborns						Term newborns								
		
Patient number	1	2	3	4	5	6	7	8	9	10	11	12	13	14	15
Clinical data															
Gestational age	31 w 3 d	35 w	30 w	36 w	35 w	36 w	38 w	39 w	39 w	40 w	40 w	37 w 3 d	38 w	42 w	39 w 2 d
Age at muscle biopsy (postnatal age)	20 d	0 d	1 m 2 w	5 d	19 d	12 d	7 d	4 d	6 d	7 d	17 d	39 d	2 m	1 m 15 d	3 m 15 d
Adjusted age at muscle biopsy	34 w	35 w	36 w	36 w	37 w	37 w	39 w	39 w	40 w	0 m 7 d	0 m 15 d	0 m 21 d	1 m 15 d	2 m	3 m 7 d
Age of death	5 w	1 d	Alive at 2 y 9 m	14 d	20 d	12 d	11 d	12 d	26 d	Alive at 7 y	Alive at 9 m	3 m 25 d	2 m 17 d	Alive at 13 y	5 m
Family history	–	Yes	No	No	No	No	–	Yes	Yes	No	Yes	Yes	No	No	–
Miscarriages	Yes	–	No	No	–	–	–	Yes	Yes	No	–	No	Yes	No	No
Delivery	Cesarean	Vaginal	Cesarean	Cesarean	Cesarean	–	Vaginal	Cesarean	Vaginal	Cesarean	Cesarean	Vaginal	–	Vaginal	–
Position	Cephalic	Pelvic	Pelvic	Pelvic	–	–	cephalic	Pelvic	cephalic	Pelvic	–	Pelvic	–	cephalic	–
Reduced fetal movements	Yes	Yes	Yes	No	Yes	–	–	–	Yes	Yes	Yes	Yes	–	Yes	–
Polyhydramnios	Yes	Yes	No	No	–	Yes	Yes	No	Yes	No	Yes	No	–	Yes	–
Oligohydramnios	No	No	Yes	Yes	–	No	No	No	No	No	No	No	–	No	–
Arthogryposis	No	–	No	–	–	–	Yes	No	–	–	No	No	–	No	–
Partial limbs retractions	Yes	–	Yes	–	–	–	–	Yes	–	–	Yes	Yes	–	Yes	–
Thin ribs	Yes	Yes	–	Yes	No	–	Yes	–	Yes	Yes	–	–	No	–	Yes
Respiratory failure	Yes	Yes	Yes	Yes	Yes	–	Yes	Yes	Yes	Yes	Yes	Yes	Yes	Yes	Yes
Ventilation assisted	Yes	Yes	Yes	Yes	Yes	–	Yes	Yes	Yes	Yes	Yes	Yes	Yes	No	Yes
Hypotonia	Yes	Yes	Yes	Yes	Yes	Yes	Yes	Yes	Yes	Yes	Yes	Yes	Yes	Yes	Yes
Wasting muscle	–	Yes	–	Yes	–	Yes	Yes	–	Yes	–	–	Yes	–	Yes	–
Facial weakness	No	–	–	Yes	Yes	–	Yes	Yes	–	–	Yes	Yes	Yes	Yes	Yes
Swallowing difficulties	–	–	Yes	–	–	–	–	–	–	–	Yes	Yes	–	No	–
Weight at birth (g) [SD]	1700 [+0.2]	2800 [+0.6]	1430 [−0.02]	2560 [−0.5]	–	–	2065 [−2.4]	3070 [−0.8]	2910 [−1.1]	2860 [−1.5]	2600 [−2.0]	3005 [−0.1]	3580 [+0.5]	4870 [+2.3]	–
Head circumference at birth (cm) [SD]	31.8 [+1.9]	–	–	35 [+1.1]	–	–	32 [−1.4]	36 [+0.9]	–	36.5 [+1.1]	37 [+1.5]	38.5 [+2.8]	37.5 [+1.8]	40 [+3.3]	–
Lengtht at birth (cm) [SD]	43 [+0.8]	–	–	–	–	–	47.5 [−1.0]	47 [−1.7]	–	–	50.5 [−0.5]	50 [+0.30]	–	60.5 [+3.5]	–
Pyloric stenosis	No	–	Yes	No	–	–	–	–	–	Yes	–	Yes	Yes	–	–
Undescended testes	Yes	No	Yes	–	–	–	Yes	–	No	Yes	–	Yes	No	–	Yes + small penis
Muscle biopsy															
Biopsied muscle	Vastus lateralis	Vastus lateralis	Vastus lateralis	Vastus lateralis	Deltoid	Deltoid	Vastus lateralis	Deltoid	Deltoid	Deltoid	Deltoid	Vastus lateralis	Vastus lateralis	Deltoid	Vastus lateralis
Type 1 fibers (%)	63	46	35	55	−	50	42	63	66	57	53	48	62	66	58
Central nuclei (%)	80	45	57	69	39	70	84	39	27	44	62	32	31	27	52
Satellite cells (%)															
By electron microscopy		−	5.65	9.40	7.38	7.84	13.79	9.85	5.88	5.28	8.49	8.65	13.96	−	−
Confocal, Pax7	15.94			8.28		8				4.39		10.35	14		11
Myosin															
Slow (%)	70			60		44				59		63	54		72
Neonatal	+			+		+				+		+	+		+
Developmental	−			−		±				−		−	−		−
RYR1															
Subsarcolemma	+			+		+				+		+	+		+
Cytoplasma	+			+		+				+		+	+		+
Perinuclear	++			++		++				++		++	++		++
DHPR															
Cytoplasma	+			+		+				+		+	+		+
Perinuclear	++			++		++				++		++	++		++
Desmin															
Subsarcolemma	++			++		++				++		++	++		++
Cytoplasma	+			+		+				+		+	+		+
Perinuclear	++			++		++				++		++	++		++
Vimentin															
Cytoplasma (%)	+			±		+				++		−	++		±
Caveolin															
Sarcolemma	+			+		+				+		−	+		+
Cytoplasma	±			±		±				±		±	±		±
Disferlin															
Sarcolemma	+			−		+				−		−	−		−
Cytoplasma	+			+		+				+		+	+		+

d, days; w, weeks; m, months; y, years; RYR1, ryanodine receptor type 1; DHPR, dihydropyridine receptor-a1subunit.

We noted the adjusted-age in “weeks” for the babies aged until 39 weeks of gestation at birth, while for the older babies born at term of 40 weeks or more than 40 weeks of gestation, the adjusted-age was noted in “days” and “months.”

**Table 2:** Molecular genetics findings

Patient	Adjusted-age	*MTM1* mutations	Exon	Notes and references
1	34 w	c.638T>C; p.Leu213Pro	8	This paper, missense mutation first described. Note 1: protein not detected on Western blot analysis.
2	35 w	c.1464-1467delACAG; p.Gln489Argfs X12	13	Patient FS30 in Buj-Bello et al. (2002).
3	36 w	c.1354-1 G>A	13	This paper.
4	36 w	c.141-144delAGAA; p.Glu48Leufs X24	4	This paper.
5	37 w	MTM1: c.137-?_(^*^3357_?)del and MTMR1: c.(?_-136)_(^*^2596_?)del	4–15	This paper. Note 2: deletion exons 4–15 of MTM1 and deletion of MTMR1. Detected in his mother's DNA.
6	37 w	c.1279delA+c.1333-1345del13	12	Patient FN86 in Buj-Bello et al. (2002).
7	39 w	c.1260-10A>G; p.FIQ420-421 ins	12	Patient JH64 in Biancalana et al. (2003).
8	39 w	c.343-2A>C	6	This paper. Note 3: Detected in his affected brother's DNA.
9	39 w	c.343-2A>C	6	Patient CU15 in Laporte et al. (1997).
10	0 m 7 days	c.526C>T; p.Gln176X	7	Patient GF36 in (Biancalana et al. (2003).
11	0 m 15 days	c.-10-?_678+?del	2–8	Patient CM73 in Laporte et al. (1997). Note 4: deletion exons 2–8 of MTM1.
12	0 m 21 days	c.205C>T; p.Arg69Cys	4	This paper.
13	1 m 15 days	c.1261C>T; p.Arg421 X	12	This paper.
14	2 m	c.535C>T; p.Pro179Ser	8	Patient EO38 in Buj-Bello et al. (2002).
15	3 m 7 days	c.1395-1397dupAAT; p.lle466dup	13	This paper.

*MTM1*, myotubular myopathy; w, weeks; m, months.

### Morphological studies

For all 15 patients, an open muscle biopsy was performed within the first few weeks of life; the age at the time of the muscle biopsy ranged from 1 day to 3 months of age. We standardized, the age of newborns as “adjusted-age” at muscle biopsy, and arranged the patients in chronological order according to the corrected-age (Table 1). The period analyzed after adjusting the age of the babies corresponds, chronologically, from 34 weeks of gestation (Patient 1) to 3 months and 7 days of life (Patient 15); this allowed us to study a specific period of early life in human patients. Eight muscle biopsies were taken from the vastus lateralis and seven biopsies were taken from the deltoid. Muscle biopsies were obtained after informed consent by their parents, and all specimens were analyzed in our research laboratory in Paris.

Histochemical analyses were performed as previously described (Bevilacqua et al. 2009). The morphometric analysis was performed separately by three different investigators. A mean of 500 muscle fibers (range 200–731) were analyzed for each specimen; four consecutive, nonoverlapping fields were counted.

### Immunohistochemistry

Frozen muscle samples from seven of the 15 patients were available for immunohistochemistry (Table 1). The immunoperoxidase techniques were performed as previously described (Bevilacqua et al. 2009). We quantified the ratio of satellite cells labeled for Pax7 to the total number of myonuclei by confocal microscopy. These studies were performed using antibodies directed against Pax7 (mouse monoclonal IgG1 SC-81648, 1/20, Santa Cruz biotechnology, Santa Cruz, CA), Antilaminin (Affinity Isolated Antigen Specific Antibody L9393, 1/50, SIGMA, St. Louis, MO), and mouse Fab (ChromPure Mouse IgG 015-000-007, 1/50, Jackson, Baltimore, MD). 4′,6-diamidino-2-phenylindole, dihydrocloride (DAPI) (1/250) stained the DNA.

### Electron microscopy

Electron microscopy studies were performed on the 13 biopsies. The total number of satellite cells was counted on 30 ultra-thin sections and nonoverlapping fields of muscle specimens for 11 of the patients (Patients 3 to 13, Table 1) by two different investigators.

### Molecular studies

All of the parents gave informed consent for the genetic analysis. Genomic DNA was extracted from blood samples by standard methods. For patients 5 and 8 the mutations were detected in the mother's and the affected brother's DNA, respectively. DNAs were studied by direct sequencing of exons and intron–exon boundaries of the *MTM1* gene as previously described (Buj-Bello et al. 1999). For patients 5 and 11, a deletion was detected and confirmed by MLPA analysis (kit P309, MRC-Holland, Amsterdam, the Netherlands) (Table 2). For patient 1, proteins were extracted from the muscle sample and Western blot studies were performed with R2630 and R2827 antibodies as previously described (Tosch et al. 2010). No sample was available for RNA studies, precluding the detection of any other intronic mutations.

### Statistical analyses

A two-way analysis of variance (ANOVA) was performed to identify possible effects of population (XLMTM patients vs. controls) and of muscle (deltoid vs. vastus lateralis) on muscle fiber size, and % of satellite cells, type I fibers and central nuclei. The meta-distributions of muscle fiber size in each population were compared using the Kolmogorov–Smirnov test. The level of significance was set at 0.05.

## Results

### Clinical data

The main symptoms during the pregnancy were reduced fetal movements, polyhydramnios, and oligohydramnios (Table 1). Common associated signs were severe hypotonia at birth and respiratory failure requiring assisted ventilation. Facial weakness and swallowing difficulties were seen in most patients. Arthrogryposis was found in only one patient. The progression of the disease was often serious, 11 of them died before 5 months of age, three patients were still alive at 2 years and 9 months (Patient 3), at 7 years (Patient 10), and 9 months of age (Patient 11). Patient 14 had a relatively good evolution.

### Chronology of the appearance of morphological features in *MTM1* patients

**Figure 1:**
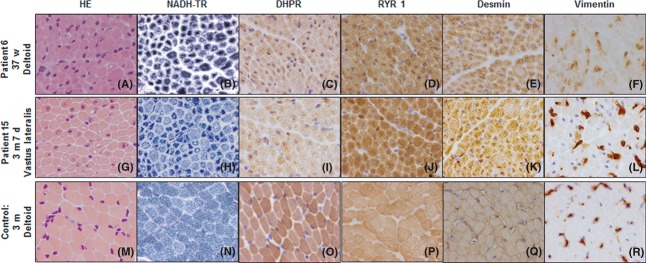
Transverse muscle sections of Patients 6 and 15 with severe X-linked myotubular myopathy showing marked variability in fiber size and the presence of numerous hypotrophic myofibers with centrally placed nuclei (haematoxylin-eosin [HE]; pictures A and G). The central area of some muscle fibers showed increased oxidative enzyme activity staining; a pale halo in subsarcolemmal regions is also observed (NADH-tetrazolium reductase [NADH-TR]; pictures B and H). Muscle sections demonstrate fibers with positive expression of either DHPR (Pictures C and I), RYR1 (Pictures D and J), or desmin (Pictures E and K) with a labeling increased in the central areas of the fibers. The immunolabeling of Vimentin is observed in some fibers (Pictures F and L). Control muscle sections (Pictures M–R). d, days; w, weeks; m, months; DHPR, dihydropyridine receptor-a1subunit; RYR, ryanodine receptor type 1.

**Figure 2:**
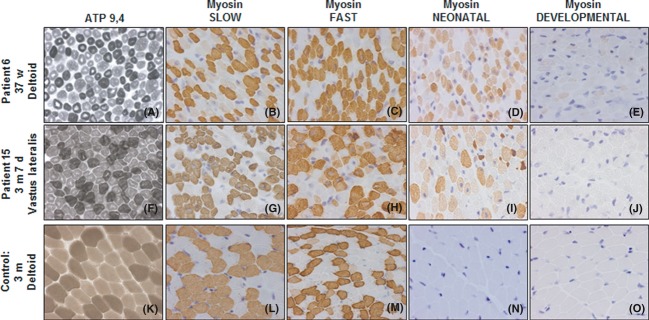
Transverse muscle sections of Patients 6 and 15 with severe X-linked myotubular myopathy. The areas surrounding central nuclei show a reduced amount of ATPase activity, in keeping with a lack of myofibrillar components in this region (ATP pH 9.4; Pictures A and F). Muscle sections treated with antibodies specific for slow (Pictures B and G), fast (Pictures C and H), neonatal/fetal (Pictures D and I), and developmental (Pictures E and J) myosin heavy chains. Control muscle sections (Pictures K–O). d, days; w, weeks; m, months.

A similar morphological pattern was observed in all muscle biopsies throughout the whole period analyzed, characterized by the presence of numerous hypotrophic fibers with central nuclei, compared to controls where the mean percentage of fibers with central nuclei was <1% (*P* < 0.0001) (Fig. 1, haematoxylin-eosin [HE]). With the oxidative staining, the fibers show a dark central region, regularly surrounded by a paler peripheral halo (Fig. 1, NADH-tetrazolium reductase [NADH-TR]). These small fibers belong to both type 1 and 2 (Fig. 2, ATPase). The mean percentage of fibers with central nuclei was 56.2% in vastus lateralis (range 31–84%) and 44.0% in deltoid (range 27–70%). In the scattered large type 1 fibers, corresponding to Wohlfart B fibers, centralized nuclei were never observed.

**Figure 3:**
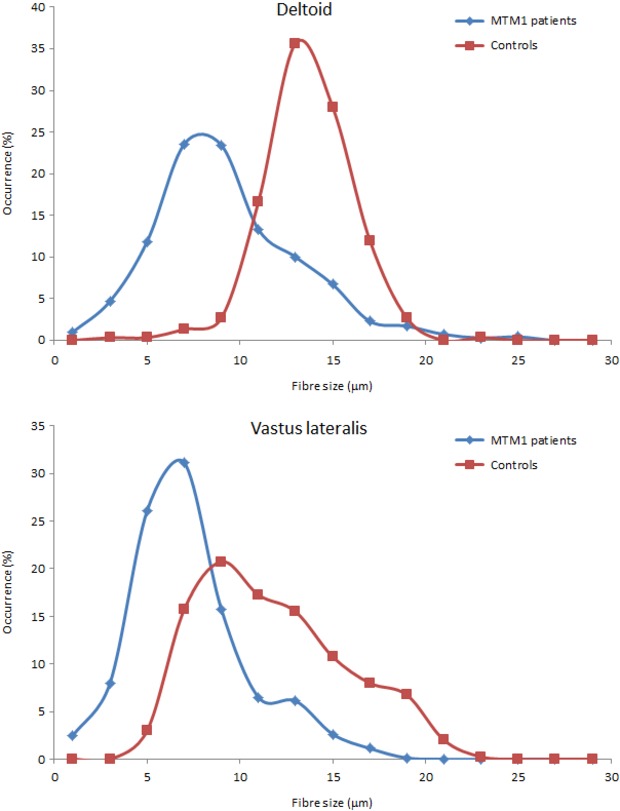
Meta-distribution of muscle fiber size observed in X-linked myotubular myopathy (XLMTM) patients and controls for the deltoid and the vastus lateralis muscles. The histograms were normalized to unit area; the amplitude in each bin is thus expressed as a percentage of occurrences. The graph shows an obvious shift in myofiber size distribution toward smaller diameters, in both vastus lateralis and deltoid, in all patients.

The diameters of muscle fibers in XLMTM patients were significantly smaller than in controls for both muscles (*P* < 0.0001) (Fig. 3). The deltoid tends to present with less small fiber size compared to the vastus lateralis (*P* = 0.078). The percentage of type I fibers was significantly higher in the patients compared to the controls (*P* = 0.002) and the deltoid presented with a significantly higher type I fiber ratio (*P* = 0.035). In the deltoid, the mean percentage of type 1 fibers was 59.2 ± 6.8% in patients and 44.9 ± 7.2% in controls. In the vastus lateralis, the mean percentage of type 1 fibers was 51.1 ± 10.0% in patients and 31.0 ± 11.6% in controls.

**Figure 4:**
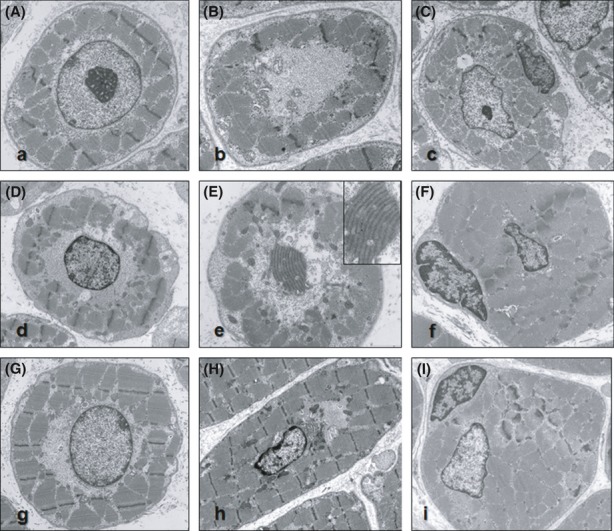
Ultrastructural analysis of muscle biopsies from Patient 4 (A and B), Patient 5 (C), Patient 7 (D and E), Patient 8 (F), Patient 12 (H), and Patient 13 (G and I). The transverse sections show myonuclei in the center of the fiber often bordered by mitochondria, glycogen, and tubular structures (Pictures A, C, D, F–I). In sections crossing the muscle fibers between two adjacent nuclei, the central area of the fibers displays a reduction of myofilaments; the space is occupied by mitochondria, glycogen, and tubular structures (Pictures B, E). Numerous cisterns corresponding to endoplasmic reticulum, triads, and Golgi complexes are found especially in the central areas of the fibers (Pictures B, E, H). Longitudinal sections for visualizing the mitochondria and glycogen that aggregate around the poles of the nucleus (E). The satellite cells are located beneath the basal lamina that surrounds each fiber. The nucleus of the satellite cells usually appears darker (with condensed chromatin) than the central nucleus in the muscle fibers (Pictures C, F, I).

Electron microscopic examination of the muscle fibers revealed central nuclei that frequently had prominent nucleoli and a substantial amount of hypercondensed chromatin. In muscle biopsies performed in infants born at term or later, the myofibrils were well structured, whereas in biopsies performed at early ages, the myofibrils appeared less compact (Fig. 4). We observed a consistent proliferation of T-tubules and sarcoreticulum cisternae in the central areas of these fibers.

### Protein analysis by immunohistochemistry

Immunohistochemical analyses of ryanodine receptor type 1 (RyR1) and dihydropyridine receptor-a1subunit (DHPR) demonstrated a marked labeling of the sarcoplasmic mesh or network mainly in the central region of the fibers (Fig. 1). These results are consistent with those observed with histoenzymological and ultrastructural studies, demonstrating the accumulation of endoplasmic reticulum, triads, Golgi apparatus, and mitochondria in the central area of the fibers.

Desmin antibodies labeled the entire cytoplasm and the staining was abnormally strong in the central region of muscle fibers. For vimentin, in both deltoid and vastus lateralis muscles, both positive and negative fibers were observed throughout the period analyzed (Fig. 1). The mean percentage of vimentin-expressing fibers was 3.5% (range 1.1–8.3%) in vastus lateralis and 5.5% (range 2.8–8.3%) in deltoid. In control patients no fibers were labeled for vimentin in biopsies performed on infants born after 36 weeks of pregnancy.

In all XLMTM biopsies, many fibers were labeled using anti–slow-myosin heavy chain (MHC) antibody, while in control muscle sections the number of fibers which expressed slow-MHC was lower (Table 1). The mean percent of slow-MHC expressing fibers was 63.6% (rate 53.8–71.6%) in vastus lateralis and 52.5% (rate 43.9–60.0%) in deltoid. With the anti–fast-MHC antibody, a number of fibers exhibited variable intensity of the stain consistent with the presence of some undifferentiated fibers. Labeling with antibody for the fetal-MHC detected fibers strongly stained, fibers weakly labeled, and fibers completely negative. Immunostaining with antibody against the embryonic-MHC was negative in all patients except for one (Fig. 2). In the control muscles most fibers were stained for the fast-MHC while the number of fibers expressing slow-MHC was less than in XLMTM patients. No antiembryonic-MHC antibody-positive fibers were detected in control muscles. With the antineonatal/fetal MHC antibody, positive fibers were detected in control muscles of youngest newborns; however, this immunoreactivity was not detected in control muscles from the older babies (id. 3 months of age).

Caveolin3 immunolabeling was essentially found at the sarcolemma in all muscle biopsies, but a weak reaction was also observed in the cytoplasm in most patients but not in controls. Dysferlin immunoreactivity was normally present at the sarcolemma in all patients; however, a diffuse labeling was also observed in the cytoplasm in some patients (Table 1).

### Satellite cell study

The number of satellite cells was counted either by confocal or by electron microscopy. Both methods were not considered different from data obtained in six subjects (Wilcoxon signed rank test, *P* = 0.893). The percentage of satellite cells was significantly lower for patients compared to controls (*P* = 0.001) and significantly lower in the deltoid compared to the quadriceps (*P* = 0.013). In the deltoid, the percentage of satellite cells was 7.33 ± 1.9% in patients and 13.0 ± 1.0% in controls. In the vastus lateralis, the percentage of satellite cells was 11.3 ± 3.6% in patients and 16.7 ± 3.4% in controls.

### Molecular data

Among the 15 patients from the present cohort, seven had small or large deletions and point mutations leading to premature truncation of the protein (Patients 2,4,5,6,10,11,13), five harbored missense or in-frame insertions or deletions of amino acids (Patients 1,7,12,14,15), and three displayed changes in the canonical splice sites (Patients 3,8,9). Patient 1 carried a missense mutation that has never been described; the pathogenicity was confirmed through a Western blot analysis showing no detectable level of myotubularin (Table 2). There was no correlation between the type of mutation and age of death.

## Discussion

We reevaluated the morphological changes in muscle biopsies in a series of muscle biopsies from infants with severe myotubular myopathy genetically characterized. We focused our chronological analysis on the early period of life (from 34 weeks of adjusted gestational age to 3 months of age). Clinically, during this early period our patients presented essentially hypotonia, generalized weakness, and respiratory failure requiring assisted ventilation (Table 1).

Regarding the evolution of the morphological changes, we would like to stress that a similar morphological pattern was observed throughout the whole period analyzed. So, we did not observe any period free of morphological abnormalities in human skeletal muscle as has been reported in animal models during early postnatal development (Buj-Bello et al. 2002; Beggs et al. 2010). The morphological signs as centralized nuclei are not present in the published mammalian models at birth, unlike in patients. Other abnormalities such as T-tubules and triads misalignment have not been extensively examined before 2 weeks old in mice. Thus, while some hallmarks of CNM are not present, additional studies are required to assess if other alterations are present in the mammalian models. In addition, the proportion of myofibers with central nuclei was high in all muscle biopsies, independently of the muscle or of the adjusted-age of the patient at the time of biopsy; consequently, we demonstrate that in humans there was no correlation between the number of myofibers with central nuclei and the age of the newborns or the type of muscle biopsied.

In all cases, the type 1 Wolfhart B fibers had a normal spatial distribution. Of note, these large type 1 fibers, descending from the first generation of myoblasts which fuse to form the primary generation muscle fibers (Butler-Browne et al. 1990; Barbet et al. 1991), always contain nuclei in a subsarcolemmal location. This suggests that the underlying defect is expressed only in muscle fibers from this second wave of myogenesis.

Immunohistochemical stains on most of the muscle samples from patients with XLMTM demonstrated a persistence of fetal-specific muscle isoforms or proteins such as desmin, vimentin, and fetal myosin heavy chain, in agreement with previous observations (Sarnat 1990; Soussi-Yanicostas et al. 1991; Sewry 1998; Romero and Bitoun 2011). However, we also show a consistent increase in the intensity of labeling with antibodies for DHPRα1s, a protein/channel of the T-tubule, and RYR1, a protein/channel of the sarcoplasmic reticulum, mainly in the central areas of the myofibers, consistent with the ultrastructural findings (Figs. 1, 4).

On ultrastructural analysis, in biopsies performed at early ages, the myofibrils appeared less compact and their structure less dense; this difference could also reflect a delay in muscle maturation. We have observed a consistent proliferation of T-tubules and sarcoreticulum cisternae in the central areas of these fibers, which substantiates the alteration defined by immunohistochemistry displaying a marked labeling mainly in the central areas of the fibers (Figs. 1, 4). This is in agreement with findings in both animal models and other forms of centronuclear myopathies, where T-tubule markers have been found to be altered (Al-Qusairi et al. 2009; Dowling et al. 2009; Romero and Bitoun 2011; Toussaint et al. 2011). T-tubule disorganization visualized by immunohistochemistry has already been described in X-linked centronuclear myopathy (XLCNM) which is in agreement with the abnormal distribution of DHPRα1 and RyR1. Moreover, we suggest that there is a more general disorganization of the membrane compartments, including the sarcoplasmic reticulum. Apart from possible functional defects arising from mispositioning of the T-tubules, these additional alterations may have a strong relevance for the pathological mechanism. The additional finding that caveolin and dysferlin were abnormally located in the cytosolic compartment suggests that additional membrane compartments are mislocalized around central nuclei or that transport of these proteins to the sarcolemma is altered. Desmin was also accumulated in the central areas of myofibers, suggesting that an alteration of the cytoskeleton is linked to the mispositioning of these diverse membranous compartments. These latter findings correlate with observations made in the *Mtm1*-null mice (Hnia et al. 2011).

The fusion of satellite cells with myofibers is a basic mechanism promoting fiber growth and hypertrophy (Relaix et al. 2006; Zammit et al. 2006; Relaix and Marcelle 2009). As myotubularin has a major role in the regulation of signaling pathways involved in growth and differentiation of muscle fibers (Razidlo et al. 2011), it seemed important to measure the number of satellite cells observed in the muscle biopsies of XLMTM infants by both confocal and electron microscopy. Interestingly, the ratio of satellite cell labeling by Pax7 to the total number of myonuclei evolves differently according to the muscle territory during the early period of life. In XLMTM patients, there were fewer Pax7-labeled satellite cells in deltoid than in vastus lateralis muscles (Table 1); in both muscles these percentages were lower than in control muscle biopsies. Our findings validate earlier studies using electron microscopy (Tomé and Fardeau 1986). This finding is extremely important as it would suggest that even at these very early stages there is a defect in production of satellite cells. Therefore, the small diameter of the muscle fibers observed in XLMTM patient biopsies could be at least partially explained by the decreased number of satellite cells. A recent study also reported a significant decrease in satellite cells in the *Mtm1*-null mice (Lawlor et al. 2012). Our findings are in stark contrast to those observed in clinical and pathological closely similar conditions such as congenital myotonic dystrophy in which the number of satellite cells is markedly increased.

Histological and structural alterations were observed in all patient biopsies at all ages investigated. This is in contrast to what has been reported for the *Mtm1*-null mice which although they reproduced the main histological signs of XLMTM, remain clinically asymptomatic during the first 3 weeks of life and only later developed a progressive myopathy (Buj-Bello et al. 2002). Moreover, Labrador retrievers with mutated myotubularin are clinically normal at birth and begin to exhibit progressive generalized muscle weakness and atrophy from 7 weeks of age (Beggs et al. 2010). This apparent discrepancy may be due to the fact that muscle maturation is achieved before birth in humans while late steps of maturation are completed within the first weeks after birth in mice and dogs.

There was no strong correlation between the type of mutation and the age of death or the muscle defects noted on biopsies. A possible explanation is that missense and truncating mutations have a similar impact on the function of the protein. Indeed, previous experiments showed that most missense mutations are linked to a large decrease in the protein level, sustaining this hypothesis (Laporte et al. 2001; Tosch et al. 2010).

In conclusion this sequential morphological study in myotubular myopathy has shown that in humans there is no period free from morphological abnormalities in the skeletal muscle which is in contrast to what has been observed in mammalian models. We have also demonstrated a more general disorganization of membrane compartments as evidenced by the presence of a significant disorganization of the cytoskeletal network, the consistent proliferation of T-tubules and the cisternae of the sarcoplasmic reticulum. These changes cannot be explained solely by a delayed maturation of the muscle fiber.
